# Changing your body changes your eating attitudes: embodiment of a slim virtual avatar induces avoidance of high-calorie food

**DOI:** 10.1016/j.heliyon.2021.e07515

**Published:** 2021-07-10

**Authors:** Riccardo Tambone, Giulia Poggio, Maria Pyasik, Dalila Burin, Olga Dal Monte, Selene Schintu, Tommaso Ciorli, Laura Lucà, Maria Vittoria Semino, Fabrizio Doricchi, Lorenzo Pia

**Affiliations:** a SAMBA (SpAtial, Motor and Bodily Awareness) Research Group, Department of Psychology, University of Turin, Turin, Italy; b NPSY-Lab. VR, Department of Human Sciences, University of Verona, Verona, Italy; c Smart-Aging Research Center, Institute of Development, Aging and Cancer (IDAC), Tohoku University, Sendai, Japan; d Department of Psychology, University of Turin, Turin, Italy; e Department of Psychology, Yale University, New Haven, CT, USA; f Department of Psychology, George Washington University, Washington DC, DC, USA; g Department of Psychology, University of Rome "La Sapienza", Fondazione Santa Lucia IRCCS, Rome, Italy; h NIT (Neuroscience Institute of Turin), Turin, Italy

**Keywords:** Body ownership, Implict bias, Food, Body image

## Abstract

The virtual-reality full-body illusion paradigm has been suggested to not only trigger the illusory ownership of the avatar's body but also the attitudinal and behavioral components stereotypically associated to that kind of virtual body. In the present study, we investigated whether this was true for stereotypes related to body size: body satisfaction and eating control behavior. Healthy participants underwent the full-body illusion paradigm with an avatar having either a larger or a slimmer body than their own, and were assessed for implicit attitudes towards body image and food calorie content at baseline and after each full-body illusion session. Results showed that the illusion emerged regardless of the avatar's body size, whereas the perceived dimension of the own body size changed according to the avatar's body size (i.e., participants felt to be slimmer after embodying their slim avatar and larger after embodying their large avatar). Crucially, we found that implicit attitudes towards food, but not those towards one's own body, were modulated by the size of the virtual body. Compared to baseline, ownership of a slimmer avatar increased the avoidance of high-calorie food, whereas ownership of a larger avatar did not induce changes. Our findings suggest that the illusory feeling of being slimmer drives also the food-related stereotypes associated with that body size, increasing the regulation of eating behaviors.

## 1 Introduction

Body ownership is the non-conceptual and perceptual status that the physical body belongs to oneself [1]. It is ubiquitous to almost any human conscious experience and, as fundamental default property of human self-awareness (“my body is always there” [2]), it is expected to deeply shape our self-identity.

Researchers have begun to employ a variety of paradigms to manipulate the properties subserving body ownership and examine how and to what extent being aware of one's own body affects human cognition and behavior. The most popular paradigm (i.e., the *rubber hand illusion*) shows that it is possible to experience a temporary feeling of ownership over a dummy hand [3, 4, 5, 6, 7, 8]. During the procedure, a life-sized fake hand is placed in front of the participant congruently with his/her body (i.e., in 1^st^ person perspective), while the corresponding real hand is hidden from the view. Then, when both the fake and real hands are synchronously touched, the illusion arises. The experience is quantified at the subjective (questionnaire answers) and behavioral (proprioceptive drift, i.e., the perceived mislocalization of one's own hand towards the fake hand) levels. Recent technological developments in immersive virtual reality have given the opportunity to induce the feeling of illusory ownership over an entire *fake* body (i.e., the full body illusion). While wearing a head-mounted display, participants see a life-sized virtual body (i.e., an avatar) that visually substitutes their real body in a first-person perspective whenever they look down towards their own body. To induce the full body ownership illusion the avatar's and participant's corresponding body part need to be synchronously touched or a motion capture system has to provide visuomotor synchrony between the avatar's and the participant's movements. It is important to emphasize that the illusory experience does not occur if the participant sees the avatar in a third-person perspective or when tactile/visuomotor stimulation is given in an asynchronous manner. Moreover, the illusory ownership of the full body can take place even if the virtual body is different with respect to participant's skin color, body height, and size. See [9, 10, 11, 12] for extensive discussions on the full body illusion paradigm.

A considerable body of research suggests that the full body illusion induces also distinct behavioral, cognitive, and emotional changes. Several studies focused on the motor domain and examined how feeling ownership of a moving avatar triggers a correspondent self-attribution of the avatar's movements and/or their properties [13, 14, 15, 16, 17, 18, 19, 20]. Other studies have investigated interpersonal relationships and the way in which illusory ownership modulates harassment, motherhood, and counseling behavior [21, 22, 23, 24]. For example, it has been shown that if a mother embodies a child avatar that receives negative maternal behavior in the virtual setting, consequently that mother increased the level of parental empathy. Similarly, the embodiment of a domestic abuser in a harassed female avatar results in an improvement of offender's ability to recognize fearful female faces [22, 23]. In a similar manner, the number of shocks delivered by male participants in a virtual Milgram scenario (i.e., obedience to authority figures) was reduced if they had previously embodied a female avatar that was harassed by a group of males [23]. Other studies have focused on how various properties of the virtual body affect unconscious implicit attitudes. For example it has been demonstrated that, in adults, the embodiment of a child avatar induces a shift of the implicit attitudes about the self towards being child-like [25, 26], whereas when the avatar is older, the negative stereotypes related to ageing significantly decreases [27, 28]. Similarly, embodying an avatar of a different race significantly reduces the implicit racial bias [29, 30]. So far, all these attitude changes at the implicit level have been interpreted in terms of *body semantics* [31]. The general idea is that during the full body illusion the brain not only triggers the illusory ownership, but it also automatically drives the attitudinal and behavioral components stereotypically associated to that type of virtual body. In other words, participants behave consistently with the representation inherently associated with the avatar's specific attributes by default.

With respect to stereotypes, those related to *body size* are among the most salient in modern western societies. Indeed, mass media and individuals' daily experiences constantly convey the pervasive message that slim individuals are happy with their own body and conduct a regulated eating behavior, whereas those overweight are seen as unhappy with their own body and often committed to unregulated eating [32, 33]. Moreover, these stereotypes are strongly rooted in the concepts of *self* and *body size* and carry an important physical signature since they are experienced through one's own body. For these reasons, they can be embodied as implicit attitudes [34, 35, 36, 37] and affect behavior within the domains of interest, namely the representation of the own body and food choices. It is worth of noticing that an excessive internalization of the ‘thin ideal’ is known to contribute to the etiology of eating disorders [38]. However, such drive can also be part of non-pathological behavior, thus preventing/reversing weight gain rather than craving a culturally fixed thin body weight, as it happens in eating disorders. Drive for thinness can subserve adaptive eating conducts that can be particularly useful in the modern obesogenic environment we live in (see [39] for an extensive discussion on this point). This idea is rather consistent with the fact that, in general, non-conscious mechanisms can be key factors in healthy behavior since, often existing choices impacting heath are not accessible to introspection [40]. Previous studies that manipulated body size by means of the full body illusion have shown that the subjective feeling of owning a virtual body emerged regardless of its visual size [41, 42, 43, 44] and that such illusory experience temporarily affected the perceived own body size in the direction of the avatar's size [41, 42, 45, 46, 47]. With respect to the impact of the avatar's size on the related implicit attitudes, however, only two studies have to date experimentally investigated it [42, 48]. Preston and Ehrsson capitalized on body satisfaction and reported that embodying a slimmer avatar increased it, but, contrary to the authors' predictions, embodying a wider body did not decrease body satisfaction. Verhulst and colleagues [48] investigated eating behavior and found that embodying a larger avatar did not activate the stereotypes associated with that size as expected (e.g., increased positive attitudes towards unhealthy food) but increased instead the perceived tastiness of a coke and the healthiness of an apple. In summary, this literature is scant/contradictory and never investigated both the stereotypes related to body size (i.e., body representation and eating attitudes) at the same time. To obtain clear evidence on the possible link between the avatar's size and the associated stereotypes, here we specifically tested whether the illusion of owning a slimmer or a larger body activated the implicit biases (body satisfaction and eating control behavior) stereotypically associated with the specific body size. To answer this question, in a within-subjects design, a group of thirty healthy women underwent two sessions of the full-body illusion paradigm in which the avatar's body size was manipulated once to be larger and once to be slimmer than their own actual body (for details, see Materials and methods section as well as Supplementary video 1). Participants' implicit attitudes towards both their actual own body and ideal body image [49], as well as towards food groups based on perceived calorie content [50] were quantified at baseline and after each full-body illusion session (i.e., within-subject design) by means of two validated and reliable [37, 51, 52] implicit association test variants (the Brief Implicit Association Test [49] and the Approach Avoidance Task [50]). We predicted that the illusory ownership of a slimmer avatar would increase both the implicit positive attitudes toward one's own body (quantified as lower discrepancy between ideal and actual body image [34, 35]) and the implicit eating control behavior (quantified as avoidance of high-calorie food [53]). On the other hand, the illusory ownership of a larger avatar was expected to increase the implicit negative attitudes toward one's own body (i.e., higher discrepancy between ideal and actual body image) along with implicit uncontrolled eating behavior (i.e., approach to high-calorie food).

Supplementary video related to this article can be found at https://doi.org/10.1016/j.heliyon.2021.e07515.

The following is the supplementary data related to this article:

Video 1_REV3**The full body illusion paradigm**. Video illustrating the experimental procedures and conditions of the virtual environment.

## 2 Results

### 2.1 Full body illusion

#### 2.1.1 Body Size Estimation Task (BSET): the estimation of the own body size changed according to the avatar's body size

**Figure 1:**
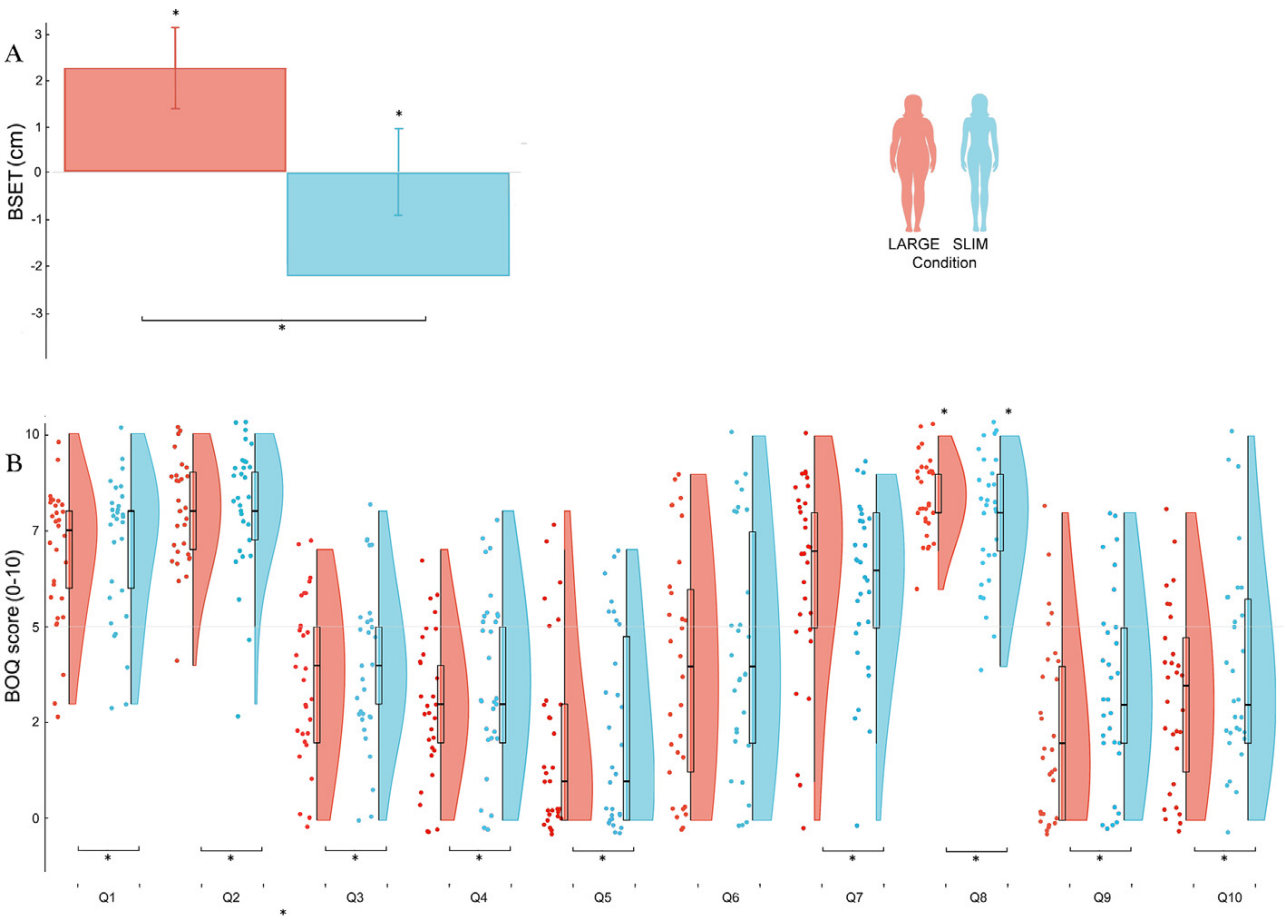
Body Size Estimation Task (BSET) and Body Ownership Questionnaire (BOQ). (A) Bar graphs show the BSET scores (mean and standard error in cm) per condition (red is the Large condition, blue the Slim condition) by using one-sample and paired t-tests. ∗ Indicates a significant (*p* < .05) difference with respect either to the point of uncertainty or to the other condition. (B) Raincloud plots show the BOQ scores (median and interquartile range) per condition (red is the Large condition, blue the Slim condition) by using one-sample and Wilcoxon signed-rank test (see Table 1 for a description of each singe question). ∗ Indicates a significant (*p* < .05) difference with respect to the point of uncertainty.

To assess the subjective perception of the own body size, we administered the BSET to each subject before (pre-illusion estimation) and after (post-illusion estimation) the full body illusion paradigm. Then, we subtracted the pre-illusion estimation from the post-illusion estimation so that negative values indicated a relative decrease in the perceived body size (i.e., participants perceived themselves slimmer) whereas positive values represented a relative increase in the perceived body size. To test changes in body size estimation we first compared the amount of change (post minus pre) for each session against zero. Results showed that the estimated body size in the Large condition (mean = 2.27, SE = ± .88) was significantly higher than zero (t(29) = 2.53, *p =* .02, d = .47, 95% CI [.47, 4.06]), whereas the one in the Slim condition (mean = -2.22, SE = ± .99) was lower (t(29) = -2.22, *p =* .03, d = .57, 95% CI [-4.25, -.18]). Additionally, not only the two conditions differed from zero but they also significantly differed from one another (t(29) = 3.08, *p* = .005, d = .86, 95% *CI* [1.46, 7.52]). These results indicate that the condition in which the avatar's body was slimmer than the participant ones induced a significant body size underestimation, whereas the one in which the avatar's body was larger induced an overestimation (see Figure 1A).

#### 2.1.2 Body Ownership Questionnaire (BOQ): the subjective experience of the illusion occurred regardless the avatar's body size

To assess the subjective experience of the full body illusion, each participant completed the BOQ after each full body illusion session. To test the presence of changes in the illusory experience, we compared the score of each of the ten questions (see Table 1) to the point of uncertainty (a score significantly higher than five represented the presence of the illusion, whereas if lower represented its absence). All the BOQ statements differed from five: Q1, Q2, Q7 and Q8 were significantly above five (all *p* < .05), whereas Q3, Q4, Q5, Q9 and Q10 were significantly below five (all *p* < .05). See Table 2 for median scores and interquartile ranges. Q6 was the only one that did not differ from the point of uncertainty in both conditions (*p* > .05; Large and Slim median = 4). These results indicate that four, out of seven, experimental questions (i.e., Q1, Q2, Q7 and Q8) displayed the illusory experience whereas all the control questions (i.e., Q4, Q5 and Q6) did not. Moreover, a between-conditions comparison (Large vs. Slim) for the four experimental questions (Q1, Q2, Q7 and Q8) revealed no significant difference for Q1 (Z = .85, *p* = .4), Q2 (Z = .52, *p* = .6) and Q7 (Z = .22, *p* = .82), whereas Q8 was significantly higher in the Large than in the Slim condition (Z = 2.02, *p* = .04, *r* = .21). These results indicate that overall, the illusory experience was not different regardless the avatar size (see Figure 1B).

**Table 1:** Body ownership questionnaire (BOQ).

Q1: How strong was the feeling that the ball you saw was directly touching you?
Q2: How strong was the feeling that you were located at some distance behind the visual image of the body that you saw?
Q3: How strong was the feeling that you were looking at someone else?
*Q4: How strong was the feeling that you had more than one body?*
*Q5: How strong was the feeling that you were drifting downwards or upwards?*
*Q6: How strongly did you feel the touch simultaneously at two locations in space?*
Q7: How strong was the feeling that the visual image of the body you saw was really you?
Q8: How strong was the feeling that the touch you felt was where you saw the ball?”)
Q9: How strong was the feeling to float in air?
Q10: How strong was the feeling that you were dissociated from your body, as if yourself and your body were in different locations?

It includes seven experimental questions (Q1, Q2, Q3, Q7, Q8, Q9 and Q10) and three control questions reported in the table in italics (Q4, Q5 and Q6).

**Table 2:** Body ownership questionnaire (BOQ).

Question	Condition	
	Large	Slim
Q1	median = 7.5, IQR = 2	median = 8, IQR = 2
Q2	median = 8, IQR = 2	median = 8, IQR = 1.75
Q3	median = 4, IQR = 3	median = 4, IQR = 2
Q4	median = 3, IQR = 2	median = 3, IQR = 3
Q5	median = 1, IQR = 3	median = 1, IQR = 4.75
Q6	median = 4	median = 4
Q7	median = 7, IQR = 3	median = 6.5, IQR = 3
Q8	median = 8, IQR = 1	median = 8, IQR = 2
Q9	median = 2, IQR	median = 3, IQR = 3
Q10	median = 3.5, IQR = 3.5	median = 3, IQR = 3.75

Median scores and interquartile ranges for all questions in the large and slim conditions.

In summary, the results of the full body illusion paradigm show that the two avatar sizes induced a different estimation of the own body size but similar subjective illusory experience.

### 2.2 Implicit attitudes

#### 2.2.1 Body Brief Implicit Association Test (BIAT): the implicit attitudes towards the actual and ideal body image did not change after the full body illusion

To assess the implicit attitudes towards the body image we administered the PBI-BIAT and the IBI-BIAT in the first session as well as at the end of each full body illusion session. The D score (i.e., index of the strength and direction of the implicit association, such that -2 reveals a preference for normal silhouettes and +2 preference for small silhouettes see [37] for details) of the first session was subtracted from each body illusion session score, so that negative numbers represented a relative decrease with respect to the baseline, and positive values indicated a relative increase. To test the presence of a significant changes in the implicit attitudes, we first compared the obtained scores for the Personal Self-identification Body Image (PBI-BIAT) and for the Ideal Body Image (IBI-BIAT) against zero. The analysis did not reveal any significant change in implicit attitudes (.12 < *p* < .94). Also, between-conditions comparison revealed no significant differences (PBI-BIAT *p* = .17; IBI-BIAT *p* = .42). These results indicate that the avatar size did not modulate the implicit attitudes towards the actual or desired body image.

#### 2.2.2 Food Preferences Approach-Avoidance Test (FP-AAT): the implicit attitudes towards food changed following the full body illusion

**Figure 2:**
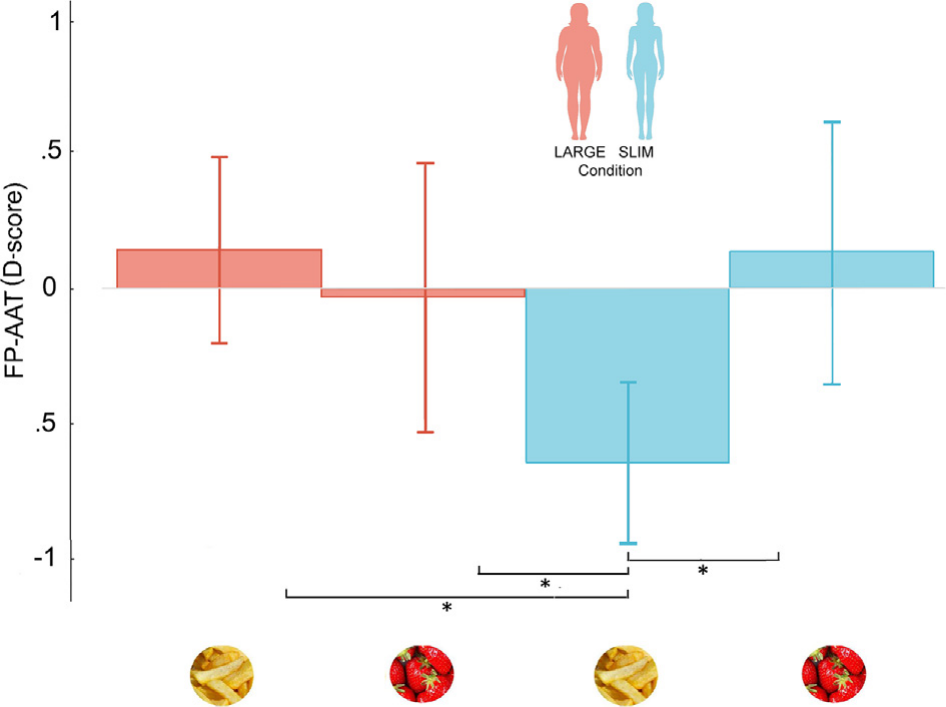
Food Preferences Approach-Avoidance Test (FP-AAT). Bar graphs show FP-AAT D-scores (mean and standard error) per condition (∗p < .05) by using one-sample and paired t-test. Red color indicates the Large condition and Blue color indicates the Slim condition.

To assess the implicit attitudes towards food, we administered the FP-AAT to each subject in the first session and at the end of each full body illusion session. The D-score obtained here represents attraction or repulsion toward low-calorie, high-calorie food, and neutral stimuli (positive values indicated approach, whereas negative values indicated avoidance). The D-score of the first session was subtracted from each body illusion session so that negative values indicated a relative decrease with respect to the baseline, and positive values indicated a relative increase. To test the presence of a significant changes in the implicit attitudes, we first compared the obtained scores against zero. This analysis revealed that when a subject embodied a slimmer avatar (Slim condition) she displayed a significant tendency to avoid high-calorie food (mean = - .64, SE = ± .3) (*t*(29) = -2.13, *p* = .04, d = .39, 95% CI = [-2.63, -1.62]) but nothing changed for low-calorie or neutral stimuli, and for any kind of stimulus in the Large condition (*p* > .05). Additionally, the score for high-calorie food in the Slim condition significantly differed (*p* < .05) from the one for low-calorie food in the in the same condition (mean = .14 SE = ± 46), and from the one for both high- (mean = -.05 SE = ± .48) and low- (mean = .14 SE = ± .34) calorie food in the Large condition; see Figure 2. These results indicate that the embodiment of a slimmer avatar increased avoidance for high-calorie food.

In summary, the results of the implicit attitudes show that the avatar size did not modulate the implicit attitudes towards their own body image but did modulate those towards food.

## 3 Discussion

We tested whether manipulating the representation of one's own body size triggered the implicit attitudes/stereotypes related to that body dimension. To answer this question a group of healthy women underwent the full-body illusion paradigm in which they embodied an avatar larger or slimmer than their own body. Participants were assessed for implicit attitudes towards actual/ideal body image and high-/low-calorie food at baseline and after each session of the full body illusion paradigm.

Firstly, the results show that our paradigm successfully induced the illusion of owning a virtual avatar. This was shown by the experimental questions assessing the subjective illusory experience targeting the participants' identification with the avatar, the estimation of the perceived intensity of the touch, and the out-of-body experience (control questions yielded no significant results). Moreover, the illusion emerged regardless the avatar's body size, i.e., the scores in the experimental questions did not differ between the two conditions. However, the perception of one's own body size differentially changed according to the avatar's body size: participants perceived themselves as slimmer after the slim condition and larger after the large condition. In agreement with previous research in healthy individuals, these findings confirm that the subjective feeling of owning a virtual body emerges regardless of its size [41, 42, 43, 44] and that such illusory experience temporarily affects the perceived own body size in the direction of the avatar's size [41, 42, 45, 46, 47].

Most importantly, we showed that implicit attitudes towards food, but not those towards one's own body, were modulated by the size of the virtual body. Specifically, ownership of a slimmer avatar increased the avoidance of high-calorie food, whereas no changes were found following the ownership of a larger avatar. How can we explain these findings? Capitalizing on evidence that the embodiment of a virtual body entails the implicit attributes associated with that body by default (and possibly activates the correspondent stereotypical behaviors [25, 26, 27, 28, 29, 30, 31]), we aimed to test whether this was true for the stereotypical behaviors linked to body size: body satisfaction and eating control behavior. As regards of the latter, it is worth noticing that the human sweet/fat preference results from an evolutionary pressure, namely selecting energy-dense foods that guarantee survival. Such innate preference, paired with the hedonic nature of food consumption (i.e., reward-driven eating) explain the reason why humans are naturally attracted to the most energy dense and palatable foods [54, 55]. However, eating too many fats/sugars negatively impacts health [54, 55]. This is particularly relevant in modern societies where the environment repletes mainly with calorically-dense palatable food cues [56] and promotes excess energy intake [57]. Beyond the fact that restricting high-calorie food is a risk factor in the development of eating disorders [58], it also entails positive impact on health [59]. In other words, under normal circumstances, the healthiness of eating habits relies on controlling the consumption of high-calorie food.

With respect to the concept of body satisfaction, it can be defined as a construct related to the representation of the own body that activates positive or negative body-related feelings [60]. Body satisfaction stems from the degree of discrepancy between the actual and the ideal body image, so that the closer is the actual body size to the ideal one, the higher is the satisfaction for the body [61]. These two stereotypes are significantly coupled: body weight, for instance, which is known to modulate the perceived healthiness of food, as well as the estimation of calorie content [62]. Most importantly, both are strictly linked to body shape and size of the own body (i.e., virtues of thinness and vices of fatness [32, 33]), so as to be interiorized as active and automatic attitudes that covertly affect behavior [34, 35, 36, 37].

Consistently with all the above-mentioned considerations, we hypothesized that ownership of a slimmer avatar would have increased the implicit eating control behavior (i.e., avoidance of high-calorie food) and the positive attitudes toward one's own body (i.e., lower discrepancy between ideal and actual body image). On the contrary, ownership of a larger body would have increased uncontrolled eating behavior (i.e., approach to high-calorie food) and implicit negative attitudes toward one's own body (i.e., higher discrepancy between ideal and actual body image). With respect to attitudes towards food, our results partially confirmed our hypothesis by showing a significant modulation only following the slim avatar condition. We argue that perceiving one's own body as slimmer might activate the concepts related to food consumption associated with that size as a default belief (i.e., controlled eating behavior). Due to the tight link between perception and action [63], this would implicitly activate the correspondent action representations within the motor system, which, in turn, would result in expressing the specific construct in terms of behavior (i.e., avoiding high-calorie food). These findings are not trivial but, rather, consistent with previous literature showing that body-related environmental cues as, for instance, commercials with slim models [64, 65] or thin human-like sculptures [66, 67] can trigger avoidance of high calorie food in absence of conscious awareness. It has been argued that these cues can, potentially, be employed to effortlessly reduce intake of high-calorie food to facilitate dieting through ‘making healthy choices easy choices’ [67]. However, we found that embodying a larger avatar did not modulate the approach towards high-calorie food. This result is in line with the finding of Verhulst and colleagues' study [48] in which the approach towards high-calorie food after the embodiment of a fat avatar was measured by means of the attitude to purchase more products with high energy intake, or saturated fat. These authors did not find any effects on purchasing behavior. Taken together, the differential effect of the body size on eating attitudes might be accounted by the fact that attitudes associated to slim bodies are inherently more adaptive than those linked to a large body. Hence, they could be evolutionary more salient and intrinsically easier to interiorize.

Although the lack of changes in body satisfaction did not confirm our hypotheses, this finding agrees with the existing literature. Indeed, the only study that so far manipulated the avatar's size and investigated its impact on the implicit attitudes towards the body in heathy participants did not report any differential effect of the size of the illusory owned body on those kind of implicit attitudes [42]. While body size-related stereotypes include implicit components, that allowed us to hypothesize about the effects at the implicit level, body satisfaction entails also strong sociocultural and cognitive components. Hence, because of the conscious components it might be difficult for a paradigm known to mainly act at the implicit level to successfully modulate body satisfaction indices, and several embodiment sessions may be necessary for it to be effective.

Before concluding, we must acknowledge some limitations, as well as possible future directions for new studies. A first limit regards the fact that we were able to collect data from thirty participants rather than form thirty-four as initially set for a standard power of .8. Despite this, such sample size resulted in a power of .7. A second limitation is related to the possible intrinsic limitations of the test-rest reliability of the implicit association test, which, despite being largely employed in research, it is still debated [68]. A third limitation concerns the fact that the changes in eating attitudes are not mediated by changes in body-related attitudes. Beyond the speculative observation that one single exposure to a different body size might not be sufficient to activate a stereotype with a higher conscious signature, this is a point that requires further investigation.

With respect to future directions, our study included solely females, however, the embodiment of a slimmer/larger body might induce different effects in a population of healthy males. Indeed, the prevalent male ideal body image is a muscular body, and a drive towards that body representation has been linked to possible phenotypes of male eating disorders (see [69] for review). Hence, it might be worth investigating whether different types of ideal body image, and their consequent experimental modulations, affect implicit eating behaviors in a similar way, for example, by associating healthier body size to more controlled eating attitudes. Perhaps the most interesting future direction is clinical in nature. It is known that eating behavior entails a fundamental automatic component, and interestingly, obese individuals display stronger approach to food as compared to normal-weight people [70]. Consistently, the approach-avoidance training, demonstrates the trainability of an avoidance reaction to high-caloric food in obese individuals [71, 72]. We therefore suggest that the full-body illusion procedure could be employed to reduce high-calorie food intake in overweight/obese population. To conclude, our results provide further understanding of the concept of embodiment and pave the way to employ the full-body illusion as a tool to promote a healthier lifestyle in overweight subjects by changing one's attitude in avoiding convenience, preprocessed and unhealthy food.

## 4 Materials and methods

### 4.1 Participants

Based on a priori power analysis conducted in G∗Power [73] for a two-tailed paired t-test with a medium effect size (d = .5) and alpha level = .05, the required sample size for reaching the power of >.8 was determined to be thirty-four subjects. Inclusion criteria were: being a female between 18 and 25 years of age, with normal weight as defined by the Body Mass Index (BMI), and without any symptoms of body representation and eating disorders as quantified by the body awareness subscale of the Body Perception Questionnaire (BPQ [74]) and the Eating Disorder Inventory (EDI [75]). Since, body size stereotyping is known to be more common in young women [32, 33]) the sample included healthy young females. All participants were recruited from a participants’ database or through flyers posted on the University website and gave their written informed consent to participate in the study (approved by the ethical committee of the University of Turin prot. n. 100960). At the end of the entire experiment, four participants were excluded from the statistical analysis because of BMI score above normal range. The final sample consisted of thirty females (age in years: mean = 21.83, SE = ± .36; education in years: mean = 16.43, SE = ± .4). Demographical and clinical data are provided in Table 3.

**Table 3:** Participants' demographical and clinical data.

ID	Age	Edu (years)	BMI	Eating Disorder Inventory (EDI)									BPQ
				Thinness	Bulimia	Body Dissatisfaction	Ineffectiveness	Perfectionism	Interpersonal Distrusts	Awareness	Maturity Fears	Total	
1	20	13	19.53	0	1	6	6	8	13	5	2	41	2.2
2	23	17	21.80	2	2	9	0	1	1	1	5	21	3.6
3	20	14	19.1	1	1	9	12	5	4	5	10	47	2.2
4	25	18	19.46	1	1	0	0	0	3	0	3	8	4.1
5	20	13	24.24	3	1	9	4	2	2	0	8	29	2.47
6	20	14	21.08	5	0	6	14	13	4	3	0	45	3.24
7	23	17	20.03	0	0	5	5	1	1	0	4	16	3.22
8	23	17	23.53	7	0	12	6	0	4	3	1	33	3.33
9	19	14	19	0	0	9	3	3	7	4	5	31	2.11
10	18	13	19.92	6	0	2	0	0	0	5	5	18	2.82
11	21	14	21.67	13	2	10	3	1	2	1	6	38	2.76
12	22	17	21.01	15	0	16	1	1	3	1	0	37	3.89
13	23	17	19.14	0	1	1	0	1	1	0	4	8	3.71
14	23	18	24.71	0	2	6	0	3	3	0	2	16	3.87
15	23	18	18.42	0	0	5	3	0	8	2	7	25	2.9
16	24	19	18.59	0	0	0	3	2	4	0	5	14	2.31
17	22	17	19.05	0	0	0	4	5	3	0	0	12	4.13
18	18	13	19.91	5	0	13	9	3	8	9	9	46	3.07
19	19	14	18.73	1	0	4	4	0	4	5	0	18	2.51
20	19	14	19.53	0	1	0	0	2	3	0	4	10	3.31
21	21	16	23.44	4	1	10	4	6	3	0	3	31	4.41
22	24	18	19.2	0	0	1	0	0	1	0	5	7	3.22
23	23	18	19.43	20	0	22	15	3	6	3	7	46	4.02
24	22	18	19.95	0	0	1	0	3	7	7	3	21	3.38
25	23	18	20.66	5	1	7	1	4	6	1	6	31	3.53
26	23	18	19.05	16	1	18	4	17	0	4	4	44	2.96
27	24	19	18.9	1	0	6	13	5	10	5	8	48	3.07
28	23	19	19.61	1	0	5	2	2	0	1	4	15	3.29
29	24	19	19.53	0	0	1	0	2	0	0	3	6	2.29
30	23	19	18.69	5	0	6	14	13	4	3	0	45	4.37

Id = patients' identification number. Edu = Education Level (years of formal education). BMI = Body Mass Index (normality ≥ 18.5 ≤ 24.99). EDI = Eating Disorder Inventory scales (normality ≤ 50). BPQ = Body Perception Questionnaire (normality ≥ 2.1 ≤ 4.4).

### 4.2 Procedure

The experiment consisted of three sessions, in a within-subjects’ design, separated by one week.

In the first session, participants signed the written informed consent, provided their demographical data, and their height and weight were measured to compute the BMI (along with their bust, waist, thigh, and hips size). The hips circumference was employed to manipulate the size of the avatar's body for the full body illusion, the other body's measures were taken to avoid drawing the participants attention to this crucial parameter. Participants completed, in a counterbalanced manner, the EDI, the body awareness subscale of the BPQ, the Personal Self-identification Body Image-BIAT (PBI-BIAT), the Ideal Body Image Brief Implicit Association Test-BIAT (IBI-BIAT [49]), and the Food Preferences Approach-Avoidance Test (FP-AAT [50]).

The second and third sessions entailed the administration of the full body illusion paradigm; in one session the participants were exposed to a slim avatar (Slim condition) and in the other session to a large avatar (Large condition). The procedure included an induction phase and then a measurement of the illusory effects with the Body Ownership Questionnaire (BOQ [76]) administered after the procedure, and with the Body Size Estimation Task (BSET [77]) administered before and after the procedure. At the end of each session, the EDI, the body awareness subscale of the BPQ, and the PBI-BIAT, and the FP-AAT were administered again. The order of the second and third session was counterbalanced among participants.

### 4.3 Assessment of eating disorders

#### 4.3.1 Body Mass Index (BMI)

It was computed with the formula kg/m^2^ where kg is a person's weight in kilograms and m^2^ is their height in meters squared.

#### 4.3.2 Eating Disorder Inventory (EDI)

It includes sixty-four statements grouped into three subscales quantfying eating disorder symptoms such as drive for thinness, bulimia, and body dissatisfaction, along with five general psychological features related to eating disorders such as ineffectiveness, perfectionism, interpersonal distrust, interoceptive awareness and maturity fears. Participants expressed their degree of agreement on a 6-point Likert scale ranging from 0 (never) to 5 (always).

### 4.4 Assessment of body representation disorders

#### 4.4.1 Body Perception Questionnaire (BPQ)

It assesses the subjective experience of the body and autonomic reactivity on five dimensions: body awareness (45 items), stress response (10 items), autonomic nervous system reactivity (27 items), stress style (12 items) and health history inventory (27 items). However, for the purpose of the study, we administered only the body awareness subscale. Participants expressed their degree of agreement on a 5-points Likert scale ranging from 1 (never) to 5 (always).

### 4.5 Full body illusion

Participants were immersed in the virtual environment with a Head Mounted Display (Oculus Rift CV1 equipped with two PenTile OLED displays with 1080x1200 pixels resolution, refresh rate at 90 Hz, field of view of 110° and 6 degrees of freedom). The scenario implemented, using the Unity3D platform, initially showed a room with a chair and a yellow ball suspended in midair. Then, a female-dressed avatar (Microsoft Rocketbox Avatar public library github.com/microsoft/Microsoft-Rocketbox) was presented from a first-person perspective in a position spatially matching the participant's physical body. The avatar's body size was manipulated by adding (Large condition) or subtracting (Slim condition) 30% of the participant's actual body size, which was measured as the distance between the hipbones outer edges. According to Provenzano and coworker's study [78], we employed such set of variation to prevent the creation of unrealistic thin bodies (particularly in the case of a low BMI) and to resemble the range of body misestimation reported in a recent metanalysis [79]. At this point, participants were asked to check whether the virtual body still overlapped their own body and, eventually, they had to adjust if needed. Then, while participants were asked to always look down at their body now substituted by the virtual body, two minutes of synchronous visuo-tactile stimulation were administered: the yellow ball rolled towards and away from the avatar's abdomen while an Arduino-controlled vibrotactile stimulator located on the participants' abdomen vibrated (at 5Hz of intensity) each time the ball collided the avatars' body (sixty times). After two minutes, the ball stopped rolling and remained suspended in midair (see Supplementary video 1), participants took off the Head Mounted Display.

#### 4.5.1 Body Size Estimation Task (BSET)

It was adapted from Keizer and colleagues [77] and measured the subjective estimation of the body size. Blindfolded participants sat on a chair with the arms stretched out along the hips without touching the body. Then, they were asked to flex the arms in front of them and adapt the distance between the palms to the perceived size of their hips. The distance between the palms was measured with a measuring tape and the procedure was repeated three times. The task was administered before and after the full body illusion paradigm.

#### 4.5.2 Body Ownership Questionnaire (BOQ)

It was adapted from Lenggenhager and colleagues [76] and included ten statements related to the various aspects of the subjective experience of the full body illusion (see Table 1). Questionnaire estimated the perceived intensity of the touch coming from the stimulation (Q1 and Q8), the identification with the avatar (Q3 and Q7), and the out-of-body experience (Q2, Q9 and Q10)*.* Three questions were not related to an actual possible illusion-induced experience and thus served as control questions (Q4, Q5 and Q6). Participants were asked to express their degree of agreement on a 10-point Likert scale ranging from 0 (lack of agreement) to 10 (full agreement). The task was administered after the full body illusion paradigm.

### 4.6 Implicit attitudes

Broadly speaking, implicit association tests allow to access thoughts that are outside conscious awareness or control because they employ pictorial/lexical speeded-RT based sorting tasks rather than direct non-speeded accuracy-based measures [80]. Measures of implicit attitudes towards the body and food have been already successfully employed in healthy population [36, 81]. Here we employed two variants of the brief Implicit Association Test [49] to investigate implicit attitudes towards the body, and the Approach-Avoidance Test [50] to examine the automatic attitudes towards different kind of food items based on perceived fat content.

#### 4.6.1 Body Brief Implicit Association Test (BIAT)

It evaluates the attitudes towards the actual (Personal Self-identification Body Image - BIAT, PBI-BIAT) or desired (Ideal Body Image Brief Implicit Association Test-BIAT, IBI-BIAT) body image (i.e., I *am*, I *want* to be, respectively) known to be a reliable measure [51]. Participants were required to press ‘I’ or ‘E’ key on a PC keyboard to classify stimuli according to a task irrelevant pairing criterion (e.g., rounded, or squared frame) rather than on the image content. Stimuli belonged to four different categories: two were concepts (i.e., normal/slim) and other two were bipolar attributes (i.e., ideal/not ideal and me/not me). One example of a response criterion is *“press ‘I’ when you see stimuli belonging to the category ‘slim’ or ‘ideal’ and press ‘E’ for stimuli of the category ‘normal’ or ‘not ideal’”*. The logic is that if two concepts were associated (e.g., ‘slim’ and ‘ideal’), reaction times should have been faster when they were assigned to the same response key as compared to different response keys. In other words, stimuli that are implicitly associated would elicit faster reaction times. The IBI-BIAT employs the images of normal and slim silhouettes of a female body for the concepts of ‘slim/normal’ and for the attributes ‘ideal/not ideal’ (i.e., perfect, better, great, attractive vs. not perfect, vs imperfect, bad, inferior, inadequate). The PBI-BIAT employs the same pictures for the concepts of ‘slim/normal’ and for the attributes ‘me/not me’ (i.e., I, me, mine, myself, vs them, others, of others). Both tests include a practice block (10 trails) followed by four consecutive blocks (20 trials in each block for a total of 80 trials) with a different pairing criterion. The crucial blocks were those with a concept and an attribute with the same key (i.e., block slim + ideal vs. normal + non ideal; block slim + not ideal vs normal + ideal). Stimuli appeared once within each block in a random order.

#### 4.6.2 Food Preferences Approach-Avoidance Test (FP-AAT)

This recognition test quantified the attraction or repulsion toward certain foods. The stimuli used, subserving implicit emotional valence (i.e., attraction or repulsion), were eight images of hypercaloric foods (e.g., pizza, French fries, chocolate), eight images of hypocaloric foods (e.g., fruits and white meat), and eight numerical digits served as neutral stimuli. For each of the three categories, half of the stimuli were presented within a round frame and the other half within a square frame. The frame shape was the target of the recognition test whereas the stimuli category was irrelevant to the task at end. Participants were asked to push the mouse away from them or pull it towards them according to the frame shape (circle or square). Since the stimulus semantic (e.g., calorie content) was associated to a specific valence (attraction or avoidance) it affected the participant response time according to whether the answer modality (i.e., pulling/pushing) to the frame shape feature (i.e., circle or square) was congruent or in incongruent with the stimulus category. For example, if participants had an implicit preference for hypercaloric foods, they should have been faster when asked to pull the mouse as compared to push it. To induce the perception of pushing, simultaneously with the action executed by the participant, the pictures progressively shrank until become a dot. Similarly, when pulling the mouse, the picture progressively grew bigger until it occupied the whole screen. When a wrong response was given, a red X appeared at the center of the screen and the stimulus remained on until the participant performed the correct action.

### 4.7 Statistical analysis

Analyses were performed using Python (version 3.8.5) with alpha set at .05. All data are presented as means and standard errors (SE) or median and interquartile range. To test the effects of the experimental manipulation, two-tailed one-sample or paired t-tests were used if the data were normally distributed, and Wilcoxon signed-rank test if the data were not. Effect sizes are indicated for significant effects, Cohen's *d* was used for parametric comparisons and matched pairs rank-biserial correlation *r* for non-parametric comparisons.

## Data accessibility

The code for the virtual setup and procedure is available from the Lead Contact on request. Raw data for each participant are available as supplementary material.

## Declarations

### Author contribution statement

Riccardo Tambone: Performed the experiments; Analyzed and interpreted the data.

Giulia Poggio, Laura Lucà, Maria Vittoria Semino: Performed the experiments.

Dalila Burin: Performed the experiments; Wrote the paper.

Olga Dal Monte, Selene Schintu, Tommaso Ciorli, Fabrizio Doricchi: Analyzed and interpreted the data; Wrote the paper.

Lorenzo Pia, Maria Pyasik: Conceived and designed the experiments; Analyzed and interpreted the data; Wrote the paper.

### Funding statement

This research did not receive any specific grant from funding agencies in the public, commercial, or not-for-profit sectors.

### Data availability statement

Data included in article/supplementary material/referenced in article.

### Declaration of interests statement

The authors declare no conflict of interest.

### Additional information

No additional information is available for this paper.
